# Label-Free Single-Cell Cancer Classification from the Spatial Distribution of Adhesion Contact Kinetics

**DOI:** 10.1021/acssensors.4c01139

**Published:** 2024-07-31

**Authors:** Balint Beres, Kinga Dora Kovacs, Nicolett Kanyo, Beatrix Peter, Inna Szekacs, Robert Horvath

**Affiliations:** † Nanobiosensorics Laboratory, Institute of Technical Physics and Materials Science, HUN-REN Centre for Energy Research, Konkoly-Thege út 29-33, Budapest H-1121, Hungary; ‡ Department of Automation and Applied Informatics, Faculty of Electrical Engineering and Informatics, Budapest University of Technology and Economics, Műegyetem rkp. 3, Budapest H-1111, Hungary; § Department of Biological Physics, Eötvös University, Pázmány Péter stny. 1/A, Budapest H-1117, Hungary

**Keywords:** resonant waveguide grating biosensor, cell type classification, phase-contrast microscope, deep learning, convolutional neural network, cell activity-based classification, single-cell selection

## Abstract

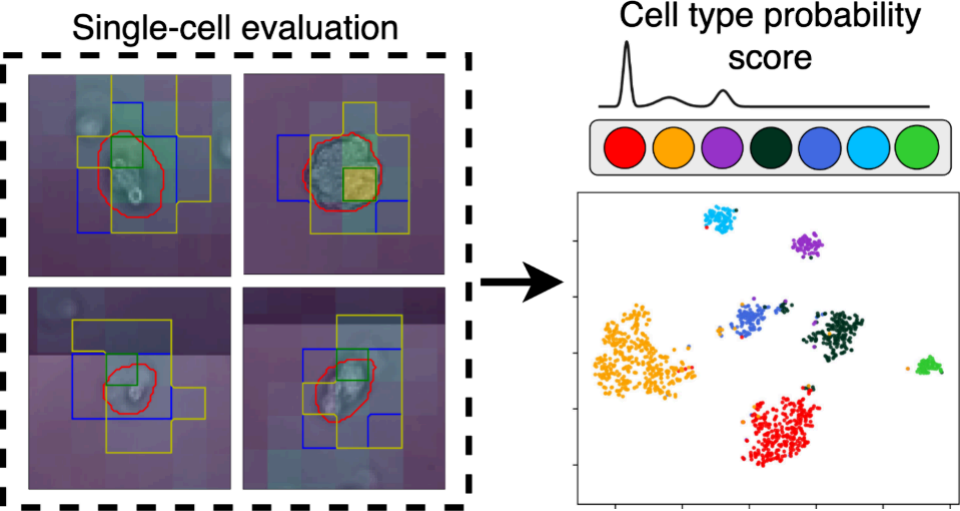

There is an increasing need for simple-to-use, noninvasive, and rapid tools to identify and separate various cell types or subtypes at the single-cell level with sufficient throughput. Often, the selection of cells based on their direct biological activity would be advantageous. These steps are critical in immune therapy, regenerative medicine, cancer diagnostics, and effective treatment. Today, live cell selection procedures incorporate some kind of biomolecular labeling or other invasive measures, which may impact cellular functionality or cause damage to the cells. In this study, we first introduce a highly accurate single-cell segmentation methodology by combining the high spatial resolution of a phase-contrast microscope with the adhesion kinetic recording capability of a resonant waveguide grating (RWG) biosensor. We present a classification workflow that incorporates the semiautomatic separation and classification of single cells from the measurement data captured by an RWG-based biosensor for adhesion kinetics data and a phase-contrast microscope for highly accurate spatial resolution. The methodology was tested with one healthy and six cancer cell types recorded with two functionalized coatings. The data set contains over 5000 single-cell samples for each surface and over 12,000 samples in total. We compare and evaluate the classification using these two types of surfaces (fibronectin and noncoated) with different segmentation strategies and measurement timespans applied to our classifiers. The overall classification performance reached nearly 95% with the best models showing that our proof-of-concept methodology could be adapted for real-life automatic diagnostics use cases. The label-free measurement technique has no impact on cellular functionality, directly measures cellular activity, and can be easily tuned to a specific application by varying the sensor coating. These features make it suitable for applications requiring further processing of selected cells.

Cell adhesion is a fundamental biological process crucial for the formation and maintenance of tissues and organs in multicellular organisms. It involves the attachment of cells to each other or the extracellular matrix through specific molecular interactions. These interactions are mediated by cell adhesion molecules, including integrins and cadherins, which play key roles in cell signaling, migration, and differentiation.^1^ Proper cell adhesion is essential for various physiological functions, such as embryonic development, immune response, and tissue repair.^2^ Dysregulation of cell adhesion can contribute to pathological conditions, including cancer metastasis and autoimmune disorders.^3,4^ Understanding the intricacies of cell adhesion mechanisms provides valuable insights into both normal physiological processes and disease states. The glycocalyx, a carbohydrate-rich layer on the cell surface, plays a crucial role in regulating cell adhesion.^5,6^ This complex and dynamic structure consists of glycoproteins and glycolipids that extend from the cell membrane.^7^ Changes in the composition or structure of the glycocalyx can have profound effects on cell adhesion, impacting processes like immune response, tissue development, and overall cellular communication.^8^ In essence, the glycocalyx serves as a dynamic interface that modulates the adhesive properties of cells, contributing significantly to the regulation of various physiological and pathological processes.

Disruptions in cell adhesion mechanisms can contribute to the development and progression of cancer.^4^ When adhesive interactions are compromised, cancer cells can gain the ability to detach from the primary tumor site, invade surrounding tissues, and eventually metastasize to distant organs.^9^

Cellular heterogeneity is a hallmark of healthy tissues and also a critical factor in understanding various biological phenomena and disease states.^10^ In the context of diseases, such as cancer, cellular heterogeneity becomes particularly pronounced, as subpopulations of cells can exhibit distinct genetic, epigenetic, and phenotypic profiles.^11,12^ This heterogeneity poses challenges in the development of effective therapeutic strategies, as treatments may be less effective against certain cell populations. Label-free measurement methods in biology have emerged as powerful tools for studying various biological processes without the need for fluorescent or radioactive labels.^13^ These techniques allow researchers to directly monitor and analyze biomolecular interactions in their native state, providing a more accurate representation of biological events thus having broad applications in drug discovery, proteomics, and the study of cellular signaling pathways, contributing to a deeper understanding of the intricacies of biological phenomena.^14^

Optical biosensors detect alterations in refractive index within a range of 100–150 nm from the sensor surface, tracking the resonance peak in response to the incident angle (utilizing optical waveguide lightmode spectroscopy (OWLS),^15^ surface plasmon resonance (SPR)^16^) or resonant wavelength/phase shift (employing resonant waveguide grating (RWG)^17^ and grating-coupled interferometry (GCI)^18^). The Epic Cardio prototype, developed by Corning Inc., is a high-throughput RWG optical biosensor designed to be compatible with standard 384-well RWG sensor microplates, making it well-suited for efficient and high-throughput biological experimentation. It has a 25 μm spatial resolution over a single 4 mm^2^ sensor surface, which, compared to its predecessor instrument, the Epic BT, is capable of accurate single-cell analysis. However, the device did not receive widespread use in the biophysical and biological community, presumably because it was not yet commercialized. It is important to emphasize that by measuring 12 sensor units in parallel, this RWG device can capture hundreds of single-cell signals simultaneously with subnanometer label-free signal resolution perpendicular to the surface. This feature distinguishes it from advanced super-resolution microscopy setups, where typically only one cell is measured at a time. Part of our aim is to provide a framework for single-cell processing using the RWG biosensor system, which could spark interest in the technology’s application and help push the method from its prototype phase to broader adoption.

RWG biosensors allow researchers to monitor the attachment and detachment of cells to and from the sensor surface.^19^ As cells adhere, alter their morphology, or undergo biochemical changes, these alterations in mass and refractive index can be precisely detected by RWG.^20^ This technology enables the quantitative analysis of cellular adhesion kinetics.

The development of high throughput label-free measurement techniques necessitates the improvement of the performance of its data processing pipelines for medical applications to achieve wide commercial use. Deep learning-based models provide greater performance for representation learning in many different medical areas. The publication of segmentation architectures such as U-Net,^30^ V-Net^35^ or convolutional neural network (CNN)- and recurrent neural network (RNN)-based models for classification have served great use in medical image processing or diagnosis. Their underlying construction enables the models to learn latent representations and features of a given data set at different levels of abstraction and apply them to specific problems in machine learning applications.

In the present work, we introduce a single-cell evaluation workflow that applies simultaneous segmentation and classification of cells based on RWG biosensor for adhesion and phase-contrast microscope for high spatial resolution data. The method applies Stringer’s et al. Cellpose^21,22^ model for segmentation retrained for our use case. Then a highly accurate localization is achieved by projecting the segmented cell areas onto the lower-resolution biosensor images. Using this we perform cell separation and classify the cell samples using deep learning-based classifier networks.^23^ The workflow was tested on data sets comprising adhesion kinetics data from seven cell types adhered on two different functionalized surfaces (fibronectin and noncoated). We also determined the optimal measurement time by testing the classification of the data sets with different temporal lengths (30, 60, and 90 min). The performance reached over 90% accuracy for both types of surfaces and achieved a maximum of 97% in the case of the fibronectin coating. We determined the 90 min-long measurements are optimal for recording cell adhesion kinetics on this surface, though performance for all timespans reached over 85%. Our method does not affect cellular functionality; therefore, it can be applied to larger evaluation pipelines where cells must be used for different experiments (immune therapy, regenerative medicine).

## Materials and Methods

All chemicals and reagents were obtained from Sigma-Aldrich Chemie GmbH (Schelldorf, Germany), unless stated otherwise.

### Cell Cultures and Cell Assays

HeLa cervical cancer cells (ECACC 9302113) were cultured in Dulbecco’s modified Eagle’s medium (DMEM, Gibco) supplemented with 10% fetal bovine serum (FBS, Biowest SAS, France), 4 mM l-glutamine, 100 U/ml penicillin and 100 μg/mL streptomycin solution.

MC3T3-E1 osteoblastic cells (ECACC 99072810) were cultured in α-modified minimal essential medium supplemented with 10% FBS (Biowest SAS, France), 2 mM l-glutamine, 100 U/ml penicillin and 100 μg/mL streptomycin solution.

LCLC-103H human lung large cell carcinoma (ACC 384), H838 human lung adenocarcinoma (ATCC CRL-5844), and MDA-MB-231 and MCF-7 breast cancer cells were cultured in DMEM supplemented with 10% FBS (Biowest SAS, France), 1% nonessential amino acids, 1 mM sodium pyruvate, and 100 U/ml penicillin and 100 μg/mL streptomycin solution.

HepG2 human hepatocellular carcinoma cells (ATCC HB-8065) were cultured in RPMI-1640 medium (Gibco) containing 10% FBS (Biowest SAS, France), supplemented with 2 mM l-glutamine, and 100 U/ml penicillin and 100 μg/mL streptomycin solution.

The cultures were maintained at 37 °C in a humidified atmosphere containing 5% CO2. For the experiments, cells were removed from the tissue culture dishes using 0.05% (w/v) trypsin and 0.02% (w/v) EDTA solution. The harvested cells were centrifuged at 200 × g for 5 min and the cell pellet was resuspended in assay buffer (20 mM 2-[4-(2-hydroxyethyl) piperazin-1-yl]ethanesulfonic acid (HEPES) in Hank’s balanced salt solution (HBSS), pH 7.4). The centrifugation was repeated two times to completely remove the cell culture media.

Cells were then counted in a hemocytometer and diluted to a final cell density of 200 cells in 25 μL of HEPES-HBSS solution.

The measurements were carried out at room temperature. Twenty-five μL assay buffer was added to 12 wells in a fibronectin or noncoated Epic 384 well cell assay microplate. The baseline was recorded for 90 min before 25 μL cell suspension was added to each well and the cell adhesion was measured for a maximum of 3 h.

The constructed single-cell database contains the data of 17 independent experiments carried out with the above-described protocols.

### Single-Cell Segmentation and Classification Workflow

In this study, we have devised a single-cell evaluation workflow which can achieve both precise cell segmentation and accurate classification of seven distinct cell types. We accomplished this using an RWG-based biosensor and a phase-contrast microscope, along with the usage and implementation of deep learning-based mask generators and classifiers.

**Figure 1:**
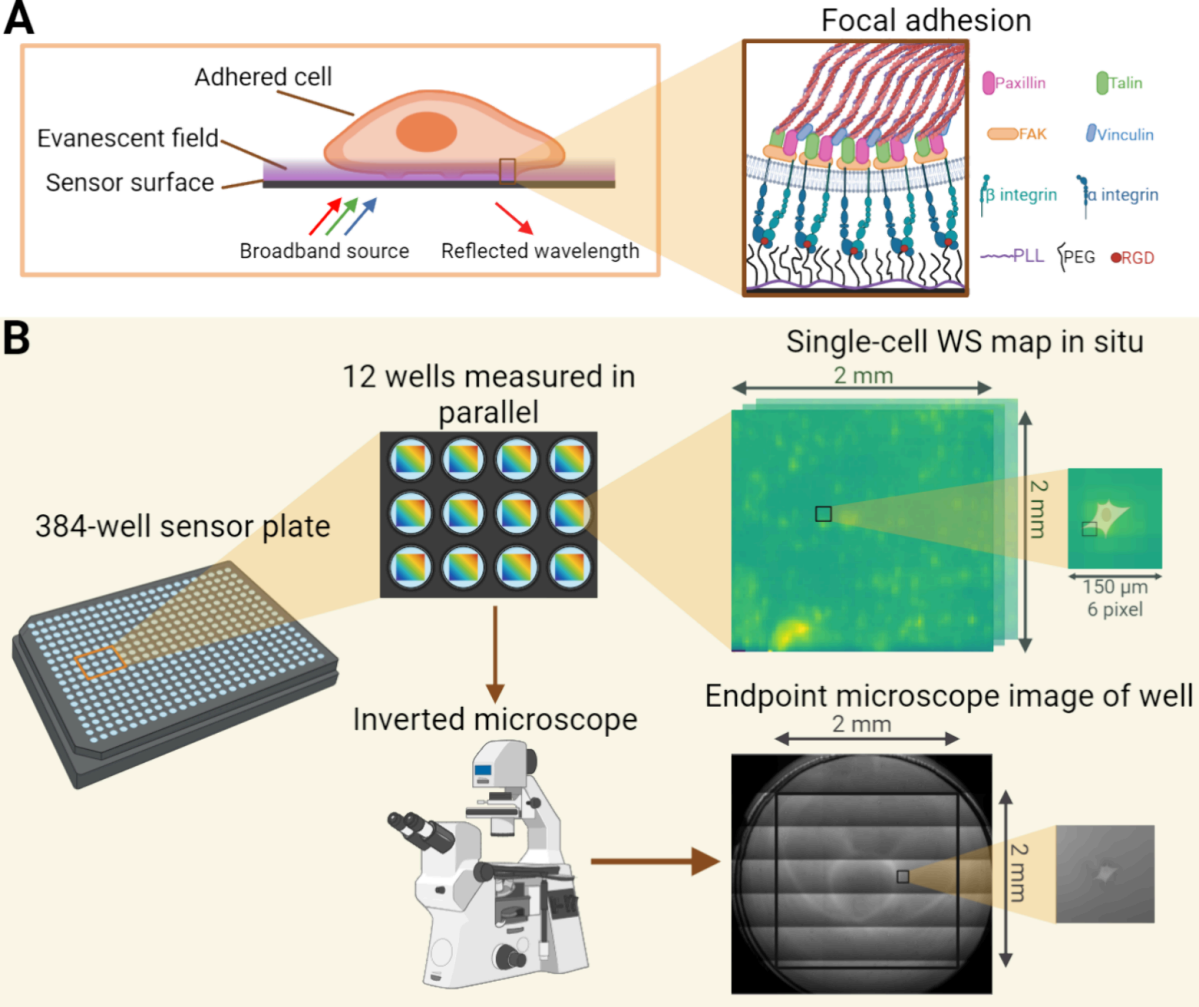
Schematic illustration of the measurement workflow, detailing the working principle of the RWG biosensor (A) and the measured adhesion complexes (B). The data is captured in two phases. First, the adhesion kinetic data is recorded using the RWG biosensor, and subsequently, each well is captured using a phase-contrast microscope for highly accurate spatial data. No changes are performed to the wells between the different measurements. Figure created using BioRender (https://biorender.com/).

The pipeline accommodates two primary types of input samples. The measurement workflow used for obtaining the data is detailed in Figure 1. First, it takes in cell adhesion kinetic data obtained through Corning Inc.’s Epic Cardio device using a 384-well microplate but only capable of measuring 12 wells parallelly. In each well there is a 2 × 2 mm wide sensor with a spatial resolution of 25 μm. Second, it involves the capture of well images via a Zeiss phase-contrast microscope after the adhesion measurements, with 20× magnification. The plate is put under the microscope with no modification to the experimental setup. By combining these two sets of samples, we can achieve highly accurate single-cell segmentation, provided that the cells remain stationary between the two data capture processes.

In the initial phase of our data processing pipeline, we acquire single-cell data using Corning Inc.’s Epic Cardio RWG biosensor, capturing spatial-temporal cell adhesion kinetic data for a maximum duration of 90 min. Following this adhesion measurement, we employ a phase-contrast microscope to capture precise spatial data of the cells. Once the measurement phase is complete, the cell adhesion data set undergoes an automatic global background correction.

Afterward, we proceed with cell localization and segmentation on the microscope data. A cell mask is generated, with each cell receiving a unique identifier within the mask. Subsequently, the two data sets are projected on the same plane to achieve precise alignment. In this semiautomated process, the scaling factor of the projection is determined based on the documented resolutions of the two devices. Then, translation properties are manually configured, and scaling errors are rectified. The output of this process is the projection properties, which are dynamically employed in the subsequent evaluation phase.

**Figure 2:**
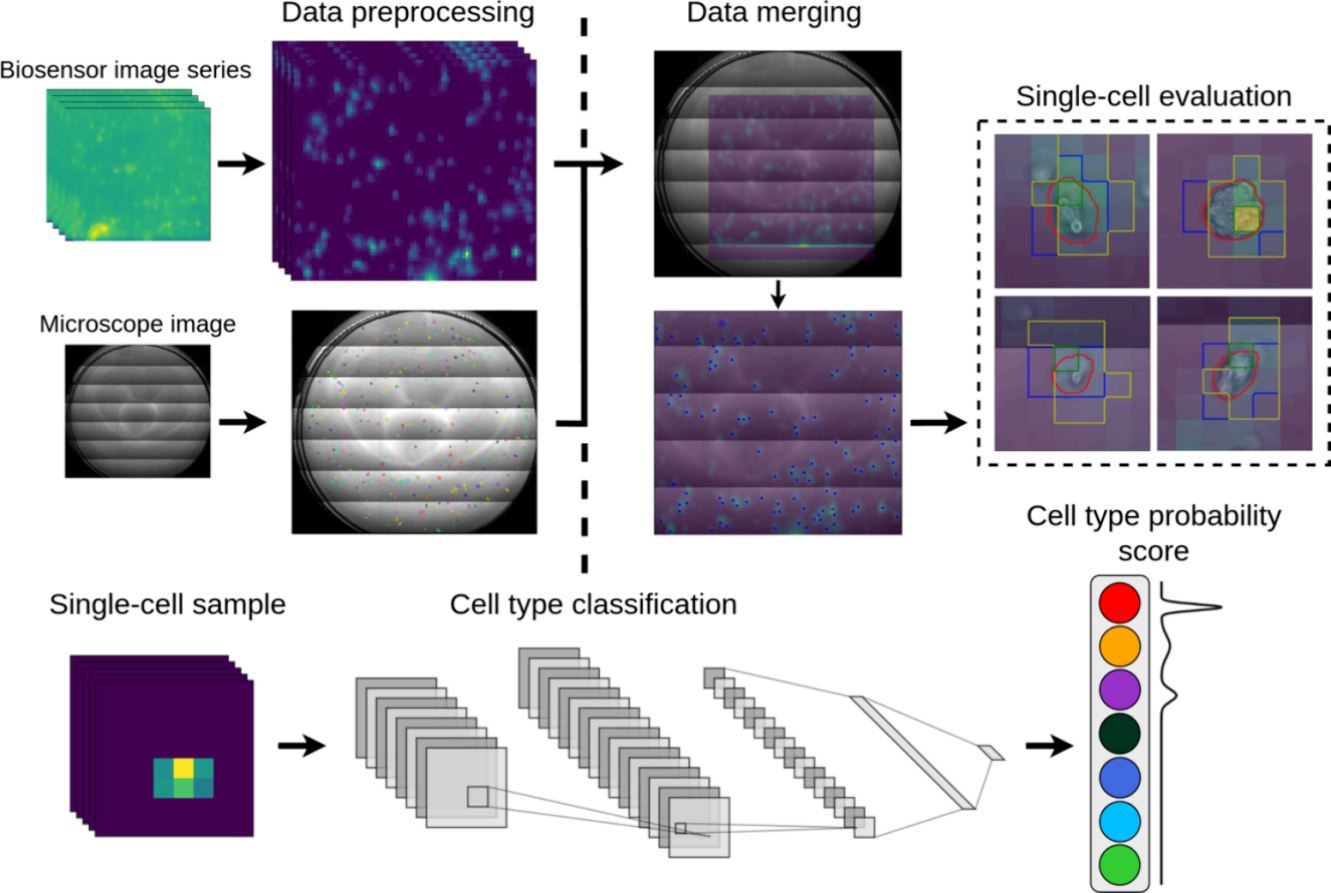
Schematic illustration of the different phases of the single-cell segmentation and classification workflow. First, the preprocessing phase happens, where both microscope and biosensor samples are preprocessed, and a segmentation mask is generated based on the microscope images. Afterward, the two samples are projected together, the images are cropped to size, and single cells separated using different segmentation strategies. Finally, samples are classified using CNN-based classifiers, which output a cell type probability vector.

Following the projection, single cells undergo assessment. Initially, cells are filtered based on their individual properties and afterward segmented according to a predefined strategy. We explored three segmentation strategies: two involving masks generated from microscope images and a standard watershed segmentation, which is widely employed for segmentation tasks. Finally, cells are exported as individual video samples for the classification phase. The full processing pipeline is illustrated in Figure 2.

In the classification phase samples are passed through CNN-based classifiers which output the probabilities for each cell type. In this context, we employed four different deep learning-based models in our cell-type classification process. We measured and evaluated model performance based on segmentation strategy and measurement timespan using data sets of seven cell types on two different functionalized surfaces.

### Single-Cell Resonant Waveguide Grating (RWG) Sensor

The Epic Cardio^31^ device from Corning Inc., USA employs the RWG (Resonant Waveguide Grating) technology for capturing optical label-free single-cell adhesion kinetics. It functions by employing a near-monochromatic infrared laser with an adjustable wavelength range of 825–840 nm. Within the waveguide, only a specific light wavelength meeting the resonance criterion can be incoupled. This criterion depends on the refractive index within the immediate vicinity (within 100–150 nm) of the sensor’s surface.

The reflected light is then directed toward a rapid CMOS (Complementary Metal–Oxide–Semiconductor) camera featuring an 80 × 80 pixel resolution and capable of swiftly scanning the entire wavelength range. The scan is conducted with a step size of 0.25 pm in under 3 s. To enhance the quality of measurements, numerous scans are gathered and averaged in the final output.

In terms of physical specifications, each sensor has dimensions of 2 × 2 mm, and the instrument has a lateral spatial resolution of 25 μm, making it particularly well-suited for detecting single cells.

### Data Preprocessing and Single-Cell Evaluation

Due to the single-cell RWG-based biosensor’s ability to capture raw wavelength-shift (WS) data for as many as 1200 individual cells during a session, we developed a semiautomated data processing pipeline to extract single-cell video data sets for subsequent classification. However, the biosensor’s resolution proves inadequate when it comes to accurately localizing and segmenting large and densely packed cell clusters. To address this limitation, we expanded the evaluation workflow to include phase-contrast microscope-based localization and segmentation, enhancing the accuracy of the identification of cell surfaces.

After measurement, the Cardio device exports a video matrix of the entire well surface, organized in a *(T, 240, 320)* configuration for 12 wells in a 3 × 4 arrangement, with *T* representing the number of temporal measurements. These matrices need to be partitioned to separate the individual wells into a *(12, T, 80, 80)* format for further use. Subsequently, the wells are trimmed to the start of the adhesion measurement phase, a calculation that can be precisely determined using the exported biosensor timeline. This is possible because there are always gaps in the timeline corresponding to when the cells are pipetted into the wells.

Following this, the wells undergo offset correction by subtracting the first frame from each time step, and outliers are removed by masking values falling outside the 3-standard deviation boundary. This step is typically necessary to exclude areas where the biosensor intersects with the well boundaries. Finally, the well requires correction for the global background noise level, achieved by selecting and subtracting the average of the background data. Since the accuracy of the background threshold can be influenced, especially at the periphery of cell regions of interest (ROIs), we implemented a pseudorandom pixel selection approach to reduce background noise. In the preprocessing stage, several background pixels are chosen, with parameters determining their distance from foreground ROIs and other selected pixels. At this stage, the foreground-background is divided by a preset 75 pm absolute wavelength shift threshold. The selection process is also centered around the cell adhesion image to mitigate potential errors at the well edges. The preprocessed samples are exported by well, ready for the projection and single-cell evaluation phases.

### Single-Cell Segmentation on the Microscope Images

To achieve precise cell localization and segmentation at a high level, we employed a multimodal strategy. In our prior research papers, we utilized a method that involved searching for local maxima while applying upper and lower thresholding to identify cells within the biosensor data. This method works well for isolated cells, but it did not effectively leverage the throughput capabilities of the Epic Cardio when dealing with larger cell clusters. In such cases, where cells have overlapping ROIs, a more precise selection of the distances between neighboring cells would be required, which would need to be tuned manually for every well.

To address this issue, after measuring adhesion with the biosensor, we used a phase-contrast microscope to capture the cell cultures. The higher resolution of the microscope enables more accurate separation of individual cells. Using this data, we projected the identified segments onto the biosensor samples to determine cell centroids and surfaces.

For parsing the microscopy images, we employed Mouseland’s Cellpose v2^21,22^ cell segmentation tool. Cellpose is a deep learning-based segmentation network that utilizes an encoder-decoder architecture to achieve state-of-the-art segmentation performance. The network outputs three separate images: a horizontal and vertical gradient of the input, as well as a background-foreground image. Combining these outputs results in a gradient vector field that encodes the position, orientation, and surface of a cell. The segmentation mask is generated from this vector field image. Cellpose v2 also offers a user-friendly tool for model development, enabling developers to easily reannotate predicted segmentation masks using an interactive user interface.

During model development, we followed an iterative approach for transfer learning the segmentation model with our data and manually annotating the ground truth images. We used Cellpose’s cyto model for pretrained model weights. We gave training and test data to our Cellpose model in an incremental fashion, increasing the data pool in small iterations. Using this approach, fewer corrections were needed to the generated masks with each iteration. In the final iteration, the model was trained using our whole microscope data set. The efficacy of this segmentation was afterward evaluated to quantify its effects on the classification loss. Of note, since the RWG biosensor had a lower resolution, heavily overlapping cells were annotated as one to leave out the issue of multicell to single sensor for further development.

### Image Projection and Single-Cell Segmentation

Due to differences in the capturing areas between the two measurement devices, we spatially aligned the two sets of samples. To address this, we developed a GUI tool in Python. The alignment happens in two steps. It works by first scaling the two samples using the precomputed scaling factor. This is calculated using the nominal properties of the devices. During the development phase, we encountered a slight variation between the theoretical and practical scaling therefore we introduced an error correction step to the alignment process. Coordinates can be manually selected on the two samples, which correspond to the same cell. Once the selection is done the program calculates the scaling and translation factors according to the following equations:
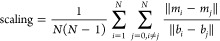

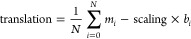
where *N* is the total number of selected coordinates on the microscope (*m*) and biosensor (*b*) samples. The scaling is corrected using the Euclidean distance ratio between vectors on the different sample planes, while the translation equates to the average difference between the vectors. The method is similar in aim to an affine transformation but leaves out the rotation and shearing operations from the projection. Once the properties were identified and the final projection was executed, we cut the size of the microscope image to match the area of the projected biosensor sample.

After achieving alignment, single cells are filtered based on both biosensor and microscope characteristics. The cells are filtered by their individual properties, such as area, maximum adhesion, and distance from the edge of the biosensor image.

We conducted evaluations using three segmentation strategies. The first strategy employed manually annotated microscope masks, and the resulting data included biosensor pixels intersecting with the masks. The second approach followed a similar technique but utilized predicted masks generated by our trained Cellpose model. The third strategy involved watershed segmentation. In this case, cell centroids were determined through a local maxima search, and regions were subsequently segmented using the watershed algorithm. The separation of foreground and background for the Euclidean distance calculation was based on the lower threshold applied during the maxima search. Single cells were automatically selected using the three strategies without any manual filtering. The cell selection happened on the projected plane, where the biosensor pixel coordinates were evaluated based on the projection properties and the final cell separation was performed on the original biosensor image. The output selections were copied onto a *(t,8,8)* video matrix and exported in *tiff* format.

### Single-Cell Classification Using Convolutional Neural Network-Based Models

After the single-cell separation, samples are preprocessed before the final classification process. Preprocessing consists of a pixel-wise standardization of the samples. Afterward, they are passed through a classifier neural network which transforms the initial three-dimensional sample into a cell probability vector. We implemented four CNN-based models: CNN, ResNet, DenseNet, and a CNN-LSTM model with added recurrent layers. These models normally accept 2D images, but we modified them to receive 3D spatial-temporal data samples.

Our CNN model simultaneously downsamples the temporal and spatial dimensions. It starts with *(t,8,8)* input dimensions and compresses the dimensions to *(**,2,2)* at the output of the feature extractor in two sequential blocks. Blocks contain 3D convolution layers with 8 and 16 channel and 3 × 3 receptive field sizes. These are followed by ReLU activation, DropOut and normalization layers. During development, we experimented with both Batch^24^ and Layer^25^ normalization. Layer normalization proved better for our use case which we applied for every normalization layer in all models. The block ends with max pooling layers which half the spatial dimensions. The first pooling layer also compresses the temporal dimension. Afterward, the output is flattened, and the classifier transforms its input in two layers to the probability vector.

We also implemented a ResNet-based^26^ model. ResNet applies a shortcut connection between stacked layers to allow the uninterrupted flow of different layers of representations through the whole classifier.^33^ This way the gradient vanishing problem can be mitigated. Our model applied basic residual blocks with *(8,16), (16,32)* channel sizes for the 3 × 3 convolutions without any bottleneck layers. The pooling and the classifier are constructed the same as it is in the previous case.

Our third model was implemented based on the DenseNet^27,34^ architecture which compared to the ResNet connects all layers by receiving the feature maps of all previous layers, concatenates them to its output and passes them to the subsequent layer.

Our final model is a CNN-LSTM-based^28^ Convolutional Recurrent Neural Network (CRNN)^29^ to try to simultaneously leverage the spatial feature extraction capability of CNN and the temporal of the Long Short-Term Memory (LSTM) layers. Our network contains three convolutional blocks, the same as described at the CNN network, but the pooling layers downsample only the spatial dimensions. Afterward, the output is passed through an LSTM block with 3 layers and 256 output size. In each case, a SoftMax operation is applied after the final layer of the classifier to produce the cell probability vector.

### Data Sets and Model Training

In this section, we provide an overview of the data sets and the training procedures employed for both the microscope segmentation and the single-cell classification models. For this research, we utilized data from 17 distinct experiments encompassing seven different cell types.

For the training of the Cellpose model, we employed a total of 175 microscope images. During the preprocessing stage, these images were divided into 2 × 2 tiles to reduce the loading time during training, resulting in a total of 700 images, which collectively contained over 13,000 cells. The data set was then split into training and validation sets in an 80–20% ratio while maintaining a uniform distribution between cell types. To achieve this, we used an oversampling data set augmentation, where the sample count of each cell type is inflated to the count of the cell type which had the most samples in the original training data set. This approach provides a data set, where in each training iteration the model can learn from cell types equally. This is performed for each cell type batch by randomly repeating samples until the batch count reaches the desired quantity. Subsequently, the models were trained over 250 epochs with a learning rate of 0.01 and a batch size of 8, utilizing pretrained cyto model weights. To optimize resource utilization, we introduced a data loading mechanism with dynamic image transformation into the existing codebase. Following the training process, masks were generated for all microscope images and exported for subsequent use in segmentation tasks.

The classification models were trained on data sets for two types of surfaces, one for fibronectin, and the other for the noncoated surface to compare the separability of the different cell classes using different functionalized surfaces. For this, we used 6 different cell types: H838, HeLa, HepG2, LCLC-103H, MCF-7, and MDA-MB-231. Also, we did a separate training with the fibronectin surface, adding a healthy MC3T3-E1(preosteoblast) cell type. The cell counts for each data set can be seen in Table 1. All measurements reached 90 min, so we capped the data sets to this timespan. To test the classification performance for different time intervals, we created three data sets with 30-, 60- and 90 min-long sample sets.

**Table 1:** Total Cell Counts of the Datasets Used for Classification^a^

cell types	coating surface	manual	predicted	watershed
H838 | HEPG2 | HeLa | LCLC-103H | MCF-7 | MDA-MB-231 (Scenario I.)	F	7330	7128	3895
	N	5195	4882	4086
H838 | HEPG2 | HeLa | LCLC-103H | MC3T3-E1 | MCF-7 | MDA-MB-231 (Scenario II.)	F	7797	7533	4355

a Two scenarios were created: Scenario I., where H838, HeLa, HepG2, LCLC-103H, MCF-7, and MDA-MB-231 were applied and Scenario II. with the added MC3T3-E1 samples. Scenario I. was tested on both fibronectin (F) and noncoated (N) coating surfaces. Separate datasets were created based on the three segmentation strategies: manual annotation, predicted masks, and watershed segmentation. Models were trained separately in three timespans: 30, 60, and 90 min-long measurements.

**Figure 3:**
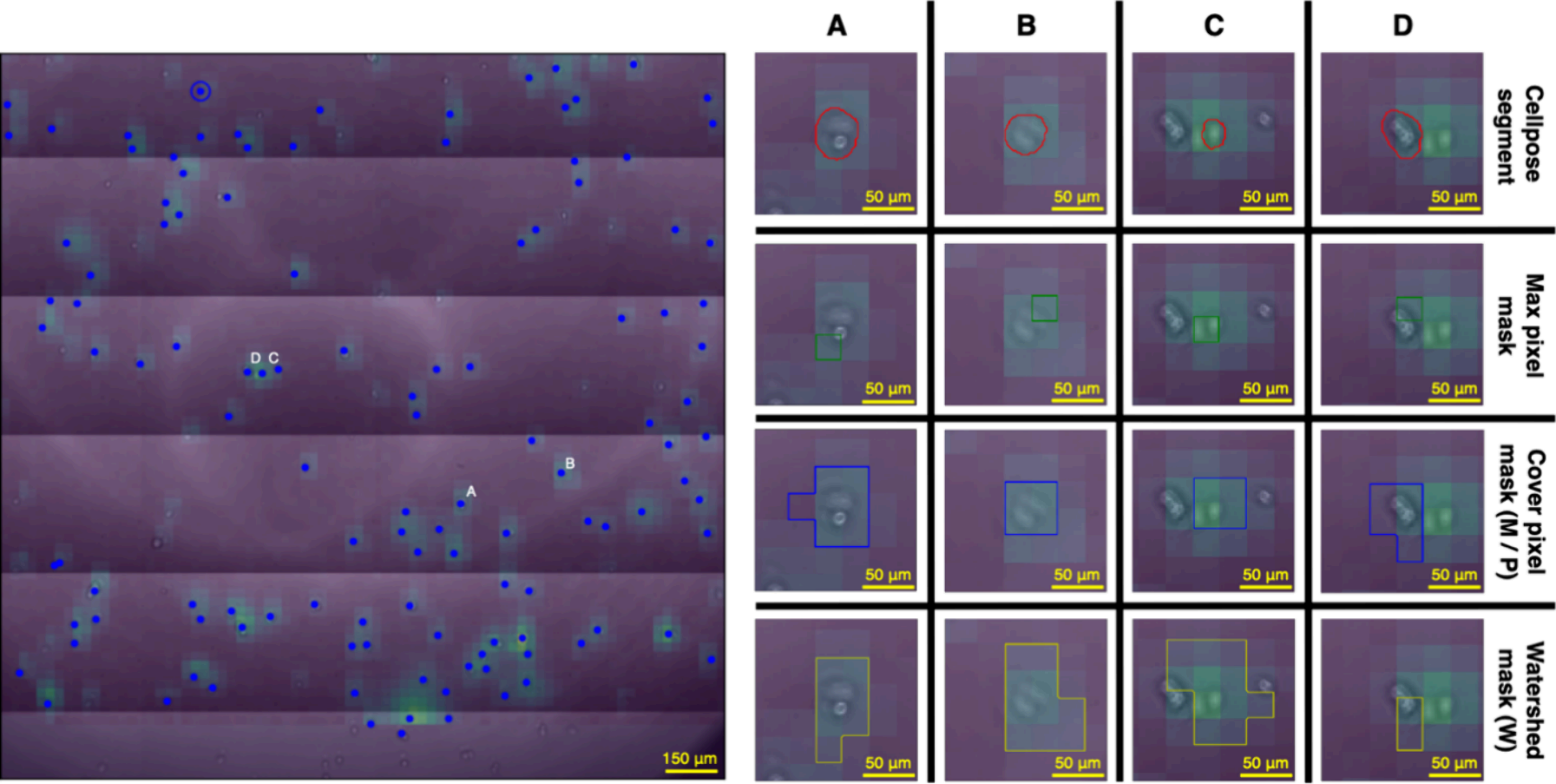
Illustration of the projected adhesion image and the single-cell segmentation strategies. The left side illustrates the aligned images. The blue markers represent the centroids of single-cell segments. We applied three strategies for segmentation. These are illustrated using four cells on the right side, which are annotated with the appropriate number on the aligned image. The blue outline (3rd row) illustrates the cover pixel-based segmentation (M), where pixels are selected if they intersect the aligned microscope segment (red outline, 1st row). This is also used for segmenting the Cellpose predicted mask (P). The yellow outline (4th row) illustrates the watershed-based segmentation (W) strategy. The maximum pixel (green outline, 2nd row) was used in our previous research to perform quantitative analysis of different cell types. Columns C and D display a segmentation scenario, where cells are grouped together. The figure illustrates a hard case of MCF-7 cells with heavy overlapping, but these provide a minority in the overall data set.

**Table 2:** Error Rates for the Segmentation Strategies^a^

		Cellpose-based segmentation			watershed segmentation	
cell types	coating surface	DE	DS_m_	DS_b_	DE	DS
H838 | HEPG2 | HeLa | LCLC-103H | MCF-7 | MDA-MB-231	F	***0.02***	***0.07***	***0.13***	0.40	0.76
	N	***0.06***	***0.06***	***0.11***	0.23	0.63
H838 | HEPG2 | HeLa | LCLC-103H | MC3T3-E1 | MCF-7 | MDA-MB-231	F	***0.03***	***0.07***	***0.13***	0.39	0.76

a Detection error (DE) shows the ratio of missed cells of a segmentation, whilst Dice-Score measures the delineation of the predicted and ground truth masks. Overall, the Cellpose-based segmentation proves to be highly accurate for both microscope segmentation shown by DS_m_ and biosensor pixel loss, DS_b_.

For classification, single-cell data sets were partitioned in a 64–16.5–16.5% ratio for training, validation and test sets. The training and optimization happened on the train and validation sets while the later performance testing and evaluation were on the test set. To counteract class imbalance, we performed the same oversampling augmentation as in the previous case. The different data sets also contain the same cell samples for maximal comparability. After the partitioning, the standardization factors (mean (μ) and standard deviation (σ)) were precomputed for later usage. The mean and standard deviation calculations were done using Welford’s online algorithm^32^ in a pixel-wise manner, where each pixel is standardized using the following equation:
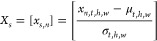
where *x* and *x*_*s*_ denote the raw and standardized samples in the *X* and *X*_*s*_ data set with *N* length and *t ϵ T*, *w ϵ W*, *h ϵ H* indices for the different axes.

Each classification model was trained for a maximum of 250 epochs using 1e-4 learning rate, 1e-5 weight decay, Adam optimizer and cross-entropy loss. To ensure that the models were not optimized on validation, or the test sets we also applied 5-fold stratified cross-validation training.

## Results and Discussion

The evaluation is presented in two phases, first the performance of the single-cell segmentation and afterward, the cell type classification is assessed. The segmentation is evaluated using two metrics: Detection Error (DE), which measures the ratio of lost cells during the segmentation for a given algorithm and Dice-Score (DS), which measures the ratio of spatial overlap between the ground truth and the predicted cell mask. Two types of DS were evaluated for the Cellpose-based segmentation: DS_m_, which measures the segmentation accuracy on the phase-contrast images and DS_b_, which shows the pixel loss ratio on the biosensor images. The metrics were evaluated for both data set scenarios and coating surfaces.

Based on the results listed in Table 2. it can be said that the Cellpose-based segmentation provides a more accurate segmentation since it shows a lower value for both metrics. For DE it stays around 5% for both coating surfaces. It also shows a lower DS rate for both scenarios. In the case of noncoated surfaces, there is a decrease in DE and DS. This is influenced by cell density in a well, the shape complexity of the individual cells and the alignment precision of the two images. As shown in Figure 3. both segmentation methods can perform with similar accuracy for less dense wells, where cells are more distant from one another, while the real benefit of our segmentation comes from applying it to more packed wells, where the cell activated areas can merge because of the lower resolution of the adhesion images. The method is still bound for cell densities by the resolution of the biosensor image. Using a phase-contrast microscope can lead to highly precise cell separation, but heavily overlapping cells are still an issue which can provide superposed adhesion signals for single sensors. To omit this issue, these cases were taken as single cells for our experiments.

**Figure 4:**
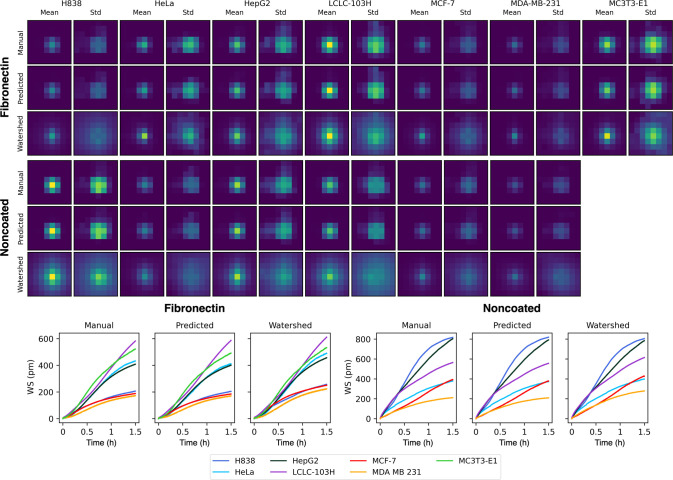
Spatial and temporal distribution of the sensor signal of the different cell types. For spatial, the kinetic average was taken of the temporal domain for 90 min measurement lengths over the 8 × 8 pixel area (200 × 200 μm^2^). Similarly, for temporal, the spatial domains were averaged to show the temporal kinetics of a given cell type.

The impact of various coating surfaces on the adhesion of different cell types in both spatial and temporal dimensions is illustrated in Figure 4. The fibronectin surface exhibits increased adhesion strength for HeLa and LCLC-103H cell lines, whereas the noncoated surface positively influences HepG2, H838, and LCLC-103H cell types. Also, on the noncoated surface, the cells appear to have a more regular shape than on the fibronectin-coated. This effect happens as the result of the cells adhering to the noncoated surface with a passive process usually taking the shape of a hemisphere, while cells can adhere to a fibronectin-coated surface through active processes forming adhesion complexes resulting in a shape characteristic of the given cell type. On the fibronectin-coated surface, cells readily find various cell adhesion motifs, such as RGD sequences.^40^ Each cell type binds to these different motifs in distinct ratios, depending on their specific integrin receptors and adhesion mechanisms. For example, some cells may preferentially bind to one type of motif more than another, leading to varied adhesion strengths and patterns.^40,41^ On the noncoated surface, there are no specific cell adhesion motifs, so cells adhere to the surface through a passive process, which does not depend heavily on the cell type, or its direct biological functionality or activity. These differences in surface properties, including the presence and variety of adhesion motifs, can lead to higher classification accuracy on the fibronectin-coated surface.

Both manual and predicted masks reveal that, on average, cells occupy a 3 × 3 pixel region. However, standard deviation images for HeLa, LCLC-103H, and MC3T3-E1 cell lines exhibit deviations. In contrast, watershed segmentation results in larger cell areas, potentially influenced by cell density. The algorithm may enlarge the area of strongly adhered cells while reducing that of weaker ones, or the overall lower precision of the background threshold may cause the foreground area to expand, resulting in more positive cell areas.

**Figure 5:**
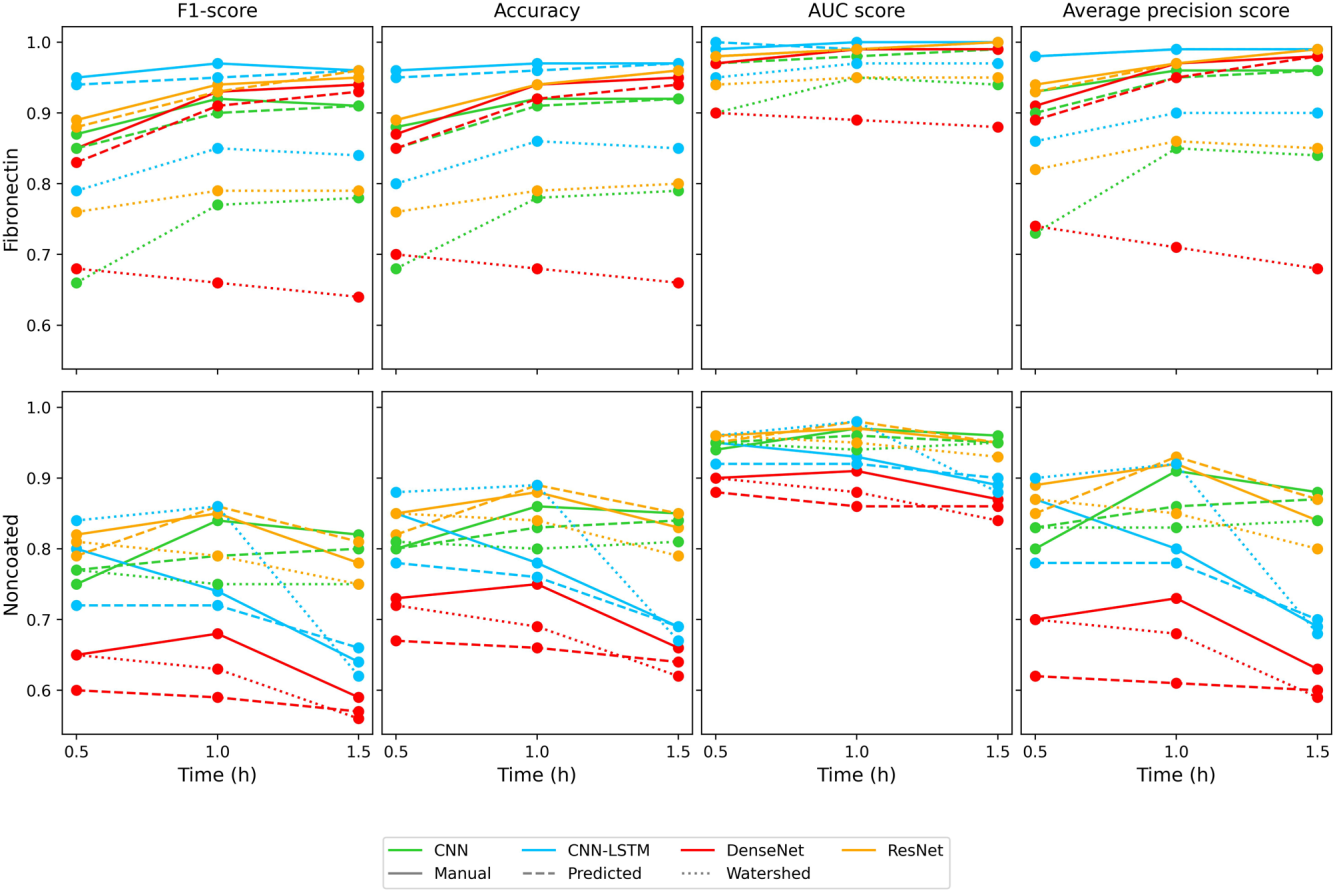
Change of the classifier evaluation metrics, trained and tested on Scenario I., for both fibronectin (upper) and noncoated (lower) surfaces.

The classification performance was evaluated using four metrics: Accuracy, F1-Score, AUC Score and AUC Precision-Recall (AUC-PR) Score. For visualizing the class separation capability of the trained models, we also applied *t*-distributed stochastic embedding(t-SNE)^36^ and Precision-Recall curve. Figure 5. shows the metrics for Scenario I. for both fibronectin and noncoated cases for every segmentation strategy.

Overall, the fibronectin coating shows a better performance across every metric and segmentation strategy compared to the noncoated parallel. There is a relative increase in the performance between the 30- and the 60 min-long data sets. The fibronectin case also shows that the classification performance for the predicted masks nearly matches the manually segmented data set which means that the deep-learning-based segmentation results in a relatively small error for the classification performance of the segmentation. The watershed-based had a worse performance, CNN-LSTM and ResNet models proving to be the best overall models for classification, but the performance is still diminished by 10–15% in this case.

The noncoated case shows a decrease in classifier performance. CNN, ResNet and DenseNet models still show a slight increase from 30 to 60 min, while CNN-LSTM shows an overall decrease in performance with increased timespans. All networks show a decrease in performance from 60 to 90 min. We investigated this phenomenon further which could be solved by increasing the network layers which could stagnate the decreasing performance after 60 min. We did not include these in the evaluation, because we wanted to compare the performance for both coating surfaces and segmentation strategies using the same networks across the board. Also, both cases show a lower variation and overall higher value in the AUC metric, which is likely caused by the imbalance in the test set, favoring the performance of cell lines which have a majority in sample count.

Tables 3 and 4 also show the metric results for the 60 min-long data sets for all networks. For fibronectin coating CNN-LSTM and ResNet models performed the best, reaching 0.95 for all metrics. The Cellpose-based segmentation proved to reach around the same level of accuracy as the manually annotated data set. The watershed-based shows a ∼ 10% decrease in metric results for both F1 and AUC-PR scores.

**Table 3:** Evaluation Metrics of the Classifiers Trained and Tested on Scenario I., Fibronectin Coating on the 60 Minute-Long Dataset

	F1-score			AUC score			AUC-PR score		
	M	P	W	M	P	W	M	P	W
CNN	0.92	0.90	0.77	0.99	0.98	0.96	0.96	0.95	0.85
ResNet	***0.94***	***0.93***	**0.79**	***0.99***	***0.99***	**0.95**	***0.97***	***0.97***	**0.90**
CNN-LSTM	***0.97***	***0.95***	**0.85**			**0.95**	***0.97***	***0.97***	**0.86**
DenseNet	0.93	0.91	0.66	0.99	0.99	0.89	0.97	0.95	0.71

**Table 4:** Evaluation Metrics of the Classifiers Trained and Tested on Scenario I., Noncoated Surface on the 60 Minute-Long Dataset

	F1-score			AUC score			AUC-PR score		
	M	P	W	M	P	W	M	P	W
CNN	**0.84**	**0.79**	**0.75**	**0.97**	**0.96**	**0.94**	**0.91**	**0.86**	**0.83**
ResNet	**0.85**	**0.86**	**0.79**	**0.97**	**0.98**	**0.95**	**0.92**	**0.93**	**0.85**
CNN-LSTM	0.74	0.72	0.86	0.93	0.92	0.98	0.80	0.80	0.92
DenseNet	0.68	0.59	0.63	0.91	0.86	0.88	0.73	0.61	0.68

For the noncoated surface, the CNN and ResNet models showed the best results. Here the Watershed-based reaches nearly the performance of the predicted data set. There is only a 2–4% difference. Compared to the fibronectin case, the noncoated surface-based classification shows a ∼ 10% decrease in performance which proves the fibronectin to be more optimal for cell type classification purposes.

**Figure 6:**
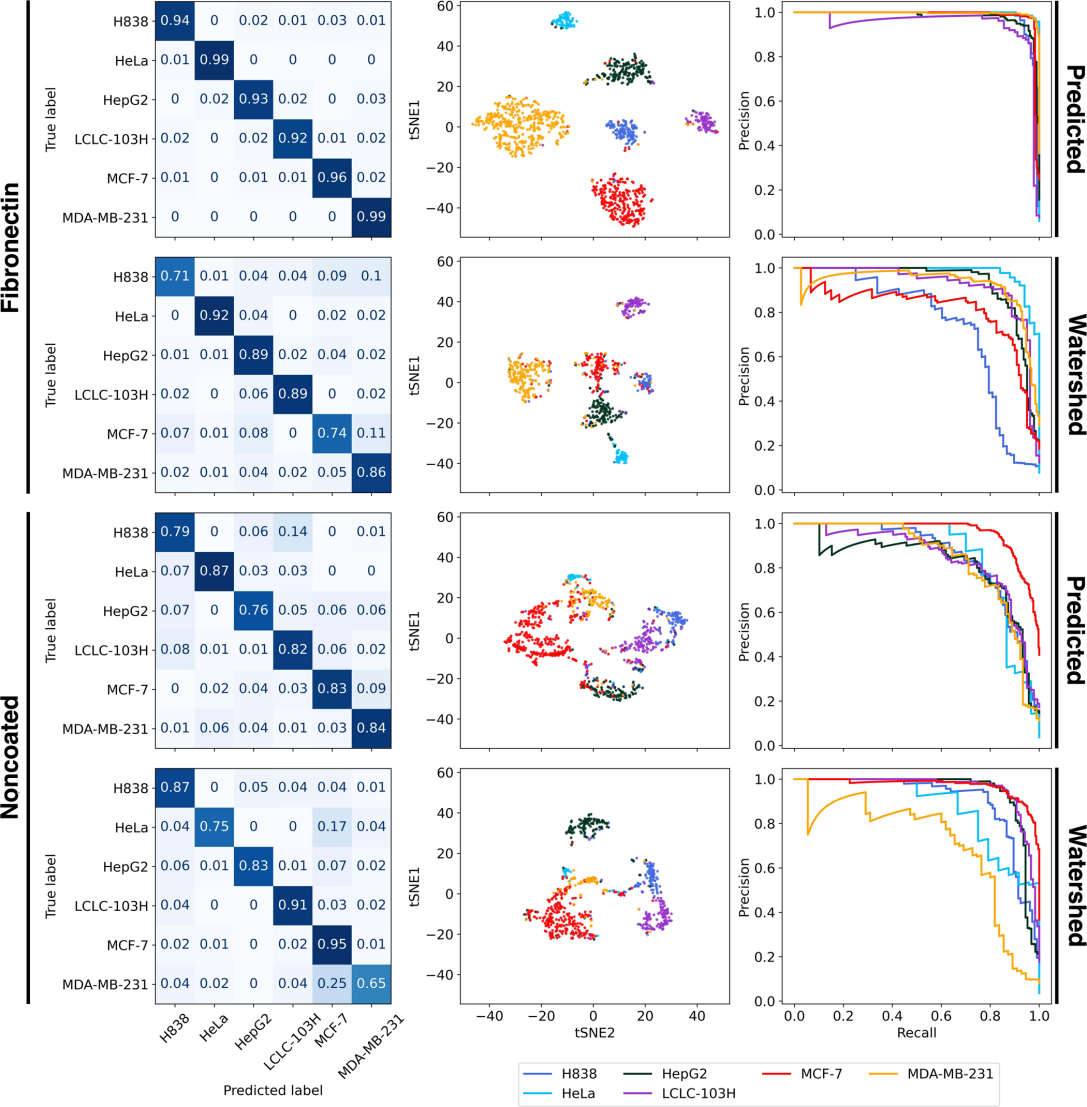
Comparative figures for network performance between coating surfaces and segmentation strategies for Scenario I. The plots show the results for the best network based on the coating surface, which is CNN-LSTM for fibronectin and ResNet for noncoated. The t-SNE plots show the spatial distribution of the output probability scores of the neural networks reduced to 2D space. Each point represents a single cell color-coded with its cell type. Good classification performance is shown by well-defined clusters of cell types with minimal intermingling. Of note, the distance between separate clusters is not necessarily informative in terms of class separability. The PR curve plots the precision against the recall for different classifier thresholds. A high area under the curve represents a lower false positive and false negative rate.

Visualizing the results based on the individual cell types, Figure 6 shows that the fibronectin case executes a near-perfect separation for the predicted data set. The confusion matrix shows an over 0.9 true positive rate for all cell types. The t-SNE plot also shows a well-defined separation between the class spaces, optimizing only a few samples to wrong cases. The PR (Precision) curve shows a significant deviation from the optimal classifier only for LCLC-103H. In comparison, the watershed case shows a worse score. Most cells reach 0.85–0.9 true positive rate. Only the H838 and MCF-7 show a value below 0.8. In this case, t-SNE shows a less defined separation between HepG2, MCF-7 and H838 cells. These cells share most of their misclassified samples. It can also be noted that H838 and MCF-7 share a substantial, 10% and 11% of their samples with the MDA-MB-231 class. In this case, the PR curve also shows a high deviation from the optimal classifier with H838 and MCF-7 showing a greater difference even from other cell types.

For the noncoated case, the ResNet model showed the best results. This coating has lower metrics scores stopping on average at 0.85 compared to the ∼0.95 for the fibronectin case. The confusion matrix shows a lower accuracy for HepG2 and H838 with H838 sharing 14% of its samples with LCLC-103H. Here the t-SNE shows higher intermingling between cell types compared to fibronectin and the PR curve also shows a lower trajectory, shared by most cell types except MCF-7 which deviates toward a more positive result. In the noncoated case, the neural network and the watershed-based segmentation produced similar results. The confusion matrix shows two notable results. MDA-MB-231 and HeLa show worse results than in the previous case sharing 17 and 25% of their samples with MCF-7. The t-SNE also shows these clusters close to each other. The PR curve also shows better results for MCF-7, HeLa and HepG2 while MDA-MB-231 shows a considerably worse trajectory.

Previously we described the classification results for both fibronectin and noncoated surfaces on the same cell pool. In the next section, we added an MC3T3-E1 cell type to the already existing data set to test how the performance is influenced by adding additional classes. This was only validated for the fibronectin surface which generally proved to show a better performance for single cell classification.

**Figure 7:**
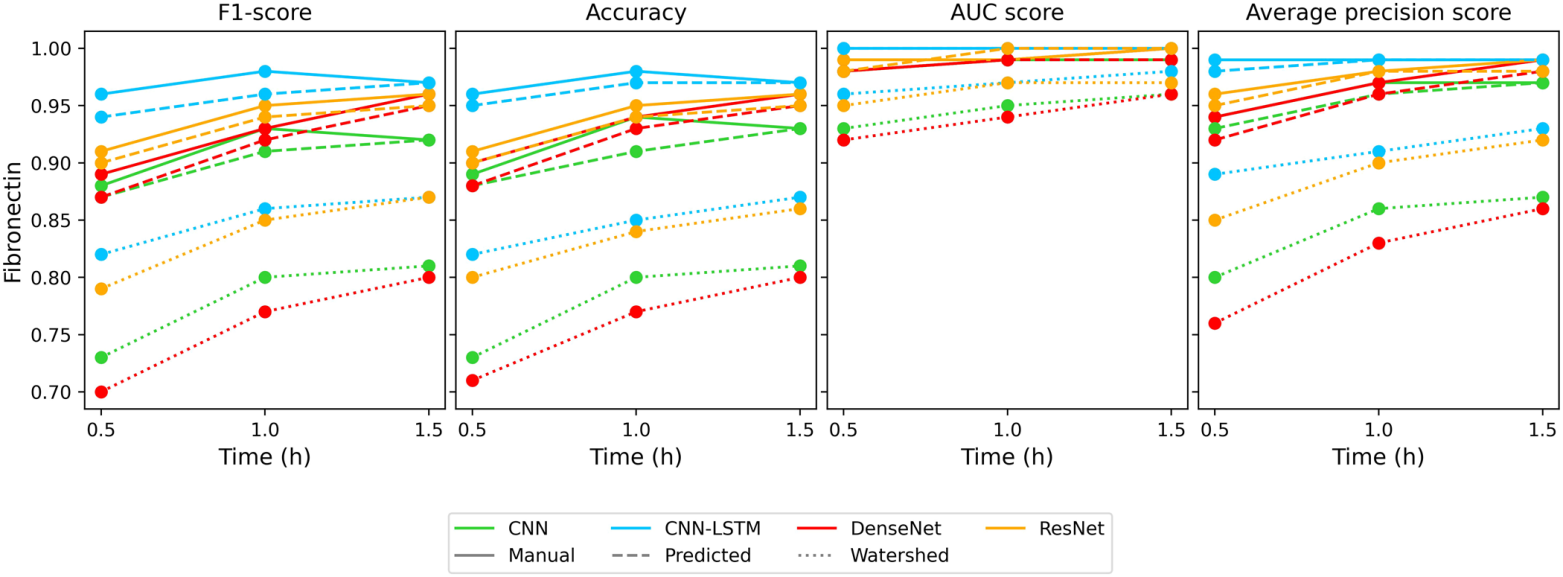
Change of the classifier evaluation metrics, trained and tested on Scenario II., for the fibronectin surface.

Similar to the previous case classification on the fibronectin plate using manual or predicted masks shows a better result. The metrics in Figure 7 and Table 5 show that the workflow is still able to achieve 0.95 F1-Score and AUC-PR score for the best models while the watershed segmentation-based data set achieves only 0.85 on F1-Score and above 0.9 on AUC-PR for 1.5-h measurement lengths. Similarly, the 30 to 60 min metric increase is the same. In this case, the CNN and CNN-LSTM models show the same decrease from 60 to 90 min which previously could be seen on the noncoated results. In this case, too ResNet and CNN-LSTM models proved to be the best classifiers.

**Table 5:** Evaluation Metrics of the Classifiers Trained and Tested on Scenario II., Fibronectin Coating on the 60 Minute-Long Dataset

	F1-score			AUC score			AUC-PR score		
	M	P	W	M	P	W	M	P	W
CNN	0.93	0.91	0.80	0.99	0.99	0.95	0.97	0.96	0.86
ResNet	***0.95***	***0.94***	**0.85**	***0.99***	***1.00***	**0.97**	***0.98***	***0.98***	**0.90**
CNN-LSTM	***0.98***	***0.96***	**0.86**	***1.00***	***1.00***	**0.97**	***0.99***	***0.99***	**0.91**
DenseNet	0.93	0.92	0.77	0.99	0.99	0.94	0.97	0.96	0.83

**Figure 8:**
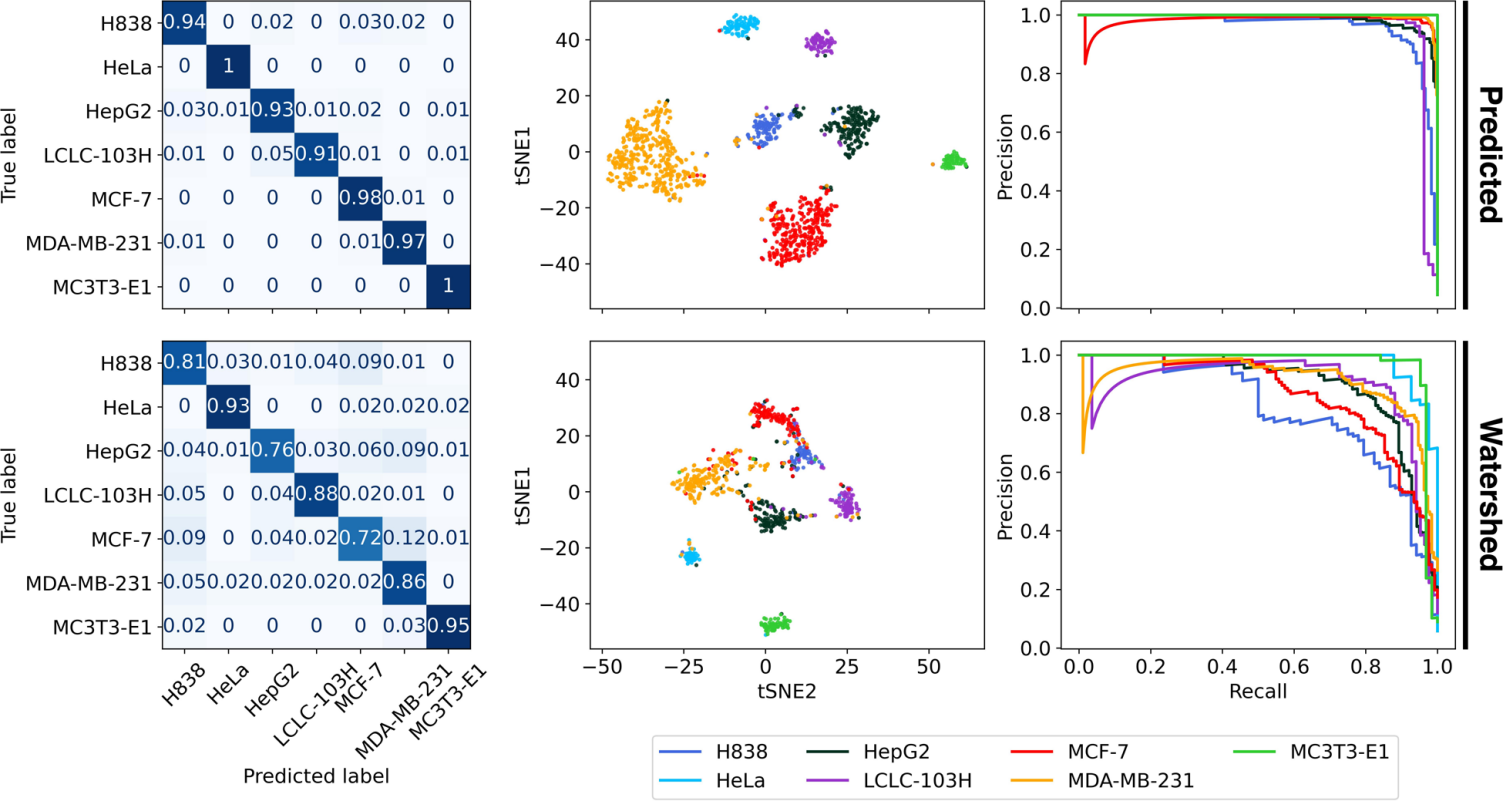
Performance of the predicted and watershed data set-based classification with the CNN-LSTM model for Scenario II. on fibronectin coating.

In both cases, the figures show a high level of accuracy (over 90% seen in Table 5). The t-SNE plots in Figure 8 show the predicted segmentation has a higher level of class separation where only outlier samples are misclassified, while the watershed data set shows that the output probability of cell types MCF-7, MDA-MB-231, HepG2 and H838 are much closer to each other. Similar to the predicted case, the PR curves show a highly accurate classification. Only the H838 and LCLC-103H cell types show a decay from the optimal classifier while the watershed case shows that HeLa and MC3T3-E1 cell types show a decay in precision.

We also performed a featured-based classification on aggregated biophysical properties of the samples in the Supporting Information using Random Forest, AdaBoost and KNeighbors classifiers.

## Conclusions

We presented a single-cell evaluation workflow which is capable of highly accurate cell segmentation and classification based on RWG biosensor and phase-contrast microscope data. We tested the method using seven different cell types and two types of surfaces. The data set comprised of over 12,000 samples across the two surfaces, recorded in 17 measurements. The classification performance reached over 95% for the fibronectin coating with an optimal measurement time between 60 and 90 min for over 90% accuracy with ResNet and CNN-LSTM models showing the best results. Since we tried to maximize classification performance while minimizing the required measurement time, we concluded, that captures capped at 90 min are enough for optimal performance for our models.

Compared to our method, state-of-the-art single-cell processing systems such as flow cytometry^37,39^ and fluorescence-activated cell sorting (FACS)^38^ provide a high throughput method for processing and sorting methods capable of processing several thousands of cells per second which can capture cell biophysical properties such as size and shape, cell–cell interactions and protein localization however our RWG biosensor-based classification method provides reliable performance in cell type identification, is completely label-free and uses a parallel capturing. It also includes temporal data of adhesion and cell migration, which can provide useful information for further analysis, and the capturing surface is experimentally tunable for better performance with new sample types and applications. Since our proposed method is a cell activity-based classification, it would have advantages in novel applications where selected cells are further processed and with a mandatory high-quality and functional activity, such as in gene and immune therapy.

Our method could still be improved by omitting the microscope image capturing after the adhesion measurement. This could be achieved by training a U-Net-based segmentation model which can automatically segment the biosensor image by using the segmented cells from the microscope images as ground truth. Developing a method which can track cell movement and changes in the cell area could also improve the characterization of different cell types. To provide adequate testing, classification experiments could be performed in wells containing multiple cell types. To achieve this in a supervised manner cell types could be labeled using different fluorescent dyes to identify them from the microscope image. Dealing with overlapping cells remains an issue. For classification purposes, these cases can be omitted, and the models can still provide reliable results. This issue could be mitigated using the simultaneous identification of multicell objects and signal normalization based on the underlying cell count. A microstructured sensor with tiny wells for each individual cell landing on the RWG surface could also solve this issue in further applications.

## Data Availability

The single-cell analysis code is available on GitHub https://github.com/Nanobiosensorics/single-cell-classification-3d and the analyzed data set can be downloaded from https://nc.ek-cer.hu/index.php/s/Gs37r3HLDacDSd5.

## Supporting Information Available

Quantifying biophysical properties of single cells; cell type characterization based on cell area, max wavelength shift (WS), and WS change rate biophysical properties; feature-based classification of the single-cell data sets; classification using RandomForest, AdaBoost, and KNeighbors classifiers based on biophysical properties (PDF)
