# Supplementary material for: Label-Free Single-Cell Cancer Classification from the Spatial Distribution of Adhesion Contact Kinetics
Source: ACS Sens. 2024 Jul 31;9(11):5815–27. doi: 10.1021/acssensors.4c01139 (PMC11590093; doi:10.1021/acssensors.4c01139)
Supplement: Supplementary file 1 — se4c01139_si_001.pdf [file se4c01139_si_001.pdf]

SUPPLEMENTARY INFORMATION

Label-free single-cell cancer classification from the spatial distribution of adhesion contact kinetics

Balint Beres[a,b], *Kinga Dora Kovacs*[a,c], *Nicolett Kanyo*[a], *Beatrix Peter*[a],

Inna Szekacs[a], *Robert Horvath* [a,*]

[a] *Nanobiosensorics Group, Institute of Technical Physics and Materials Science, Centre for Energy Research, Konkoly-Thege út 29-33, H-1121 Budapest, Hungary*

[b] *Department of Automation and Applied Informatics, Faculty of Electrical Engineering and Informatics, Budapest University of Technology and Economics, Műegyetem rkp. 3., H-1111 Budapest, Hungary*

[c] *Department of Biological Physics, Eötvös University, Pázmány Péter stny. 1/A H-1117 Budapest, Hungary*

**Email:*
horvath.robert@ek.hun-ren.hu

Quantifying biophysical properties of single cells

We aggregated several biophysical properties from the exported single-cell samples to analyze differences between cell types using quantitative methods. These properties are the cell area, measured by the non-zero-pixel count of sample frames averaged on the temporal dimension, maximum wavelength shift, which is the max sensor value of a sample, and saturation speed, quantified by the speed with which the mean adhesion kinetic signal of a single cell reaches from the 5th percentile value to the 95th percentile value. These parameters are shown per cell type and segmentation types in **Figures S1** and **S2** for both fibronectin and noncoated surface datasets.

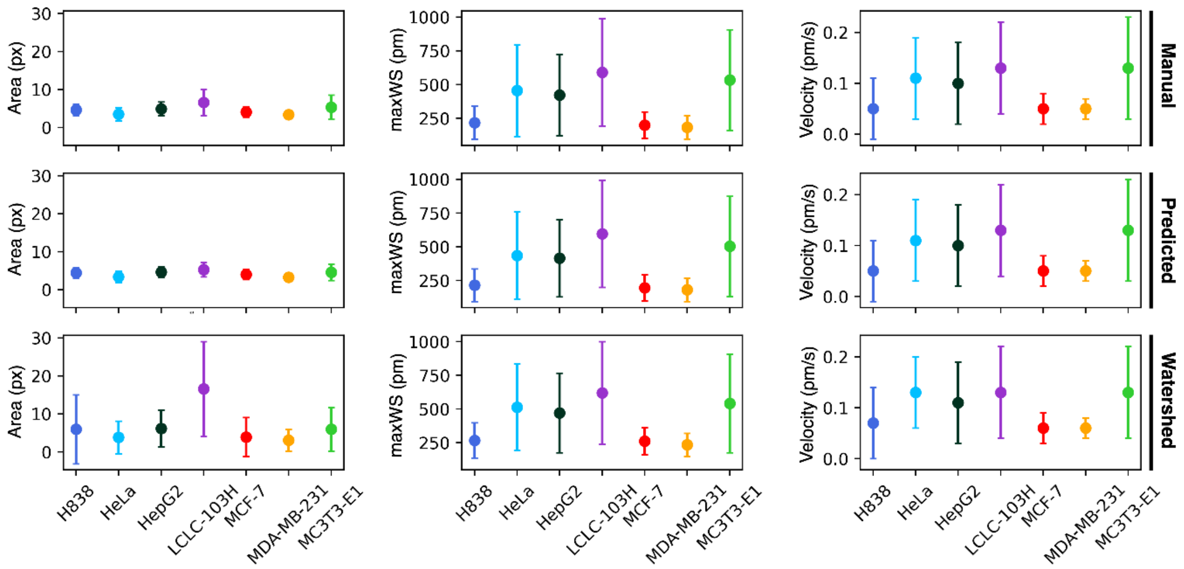

Figure S1. *Shows the mean and standard deviation for the aggregated single-cell properties per cell type on the fibronectin-coated surface. The properties were evaluated for all three segmentation types.*

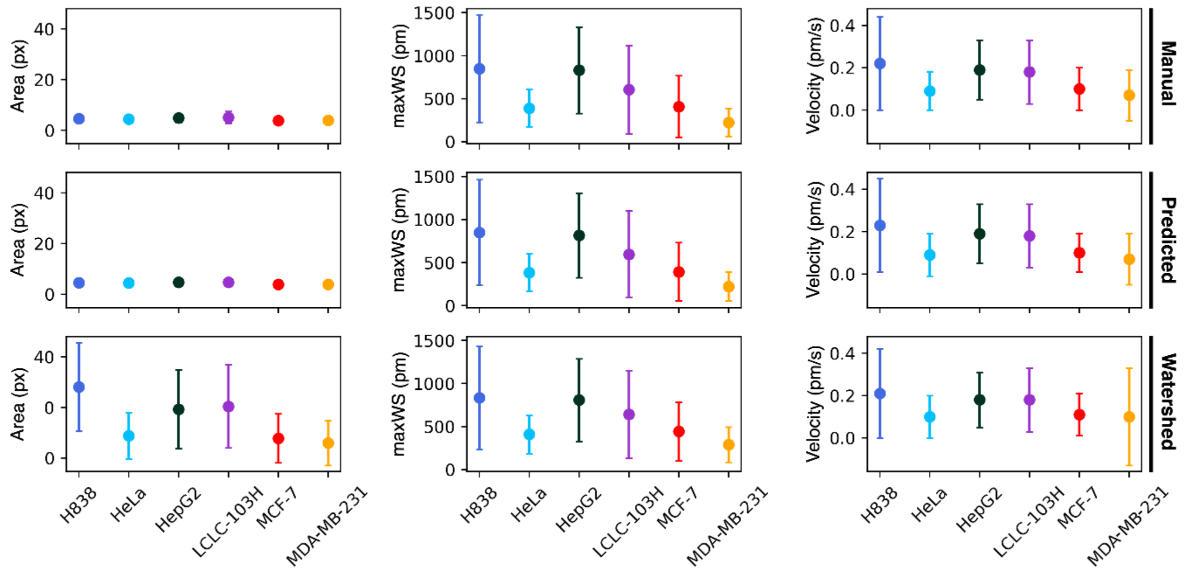

Figure S2. *Shows the mean and standard deviation for the aggregated single-cell properties per cell type on the noncoated surface. The properties were evaluated for all three segmentation types.*

Both figures show that there is a high similarity between all segmentation types for *maxWS* and *Velocity* properties, however there is a large deviation between the Cellpose-based and Watershed cell areas. The Watershed shows a great difference in both mean and standard deviation. This is also illustrated in Figure 4, where the spatial signal distribution shows a higher variation in the object size. Since both *maxWS* and *Velocity* properties do not show a significant change, it can be concluded that the Watershed segmentation adds extra pixels to the cell area which are low activation and can pertain to the background noise.

Feature-based classification of the single-cell datasets

In this section, we perform classification using the biophysical properties to see whether cell types are determinable using predefined cell characteristics. The dataset partitioning and the preprocessing steps are the same as described in the *Datasets and model* training section. The evaluation metrics were used as in the evaluation of the neural network-based classifiers. **Table S1-3** show the classifier performance on the test sets.

	F1-Score			AUC Score			AUC-PR Score		
	M	P	W	M	P	W	M	P	W
RandomForest	0.33	0.34	0.39	0.71	0.72	0.74	0.33	0.31	0.38
AdaBoost	*0.32*	*0.31*	0.35	*0.73*	*0.74*	0.70	*0.31*	*0.31*	0.33

KNeighbors	*0.30*	*0.27*	0.29	*0.65*	*0.64*	0.64	*0.25*	*0.24*	0.27

Table S1. *Shows the evaluation metrics of the classifiers trained and tested on Scenario I., fibronectin coating on the 60-minute-long dataset.*

	F1-Score			AUC Score			AUC-PR Score		
	M	P	W	M	P	W	M	P	W
RandomForest	0.35	0.31	0.32	0.74	0.72	0.73	0.36	0.33	0.33
AdaBoost	*0.28*	*0.24*	0.29	*0.72*	*0.70*	0.66	*0.28*	*0.26*	0.24
KNeighbors	*0.26*	*0.25*	0.30	*0.63*	*0.62*	0.66	*0.23*	*0.23*	0.25

Table S2. *Shows the evaluation metrics of the classifiers trained and tested on Scenario I., noncoated coating on the 60-minute-long dataset.*

	F1-Score			AUC Score			AUC-PR Score		
	M	P	W	M	P	W	M	P	W
RandomForest	0.32	0.31	0.35	0.73	0.71	0.74	0.29	0.27	0.34
AdaBoost	*0.26*	*0.26*	0.30	*0.73*	*0.73*	0.69	*0.26*	*0.27*	0.27
KNeighbors	*0.25*	*0.24*	0.27	*0.64*	*0.63*	0.65	*0.22*	*0.21*	0.23

Table S3. *Shows the evaluation metrics of the classifiers trained and tested on Scenario II., fibronectin coating on the 60-minute-long dataset.*

Overall, the performance is heavily diminished when using this classification approach. This is to be expected since we assumed that the relevant features, which can be used for separating cell types, will be in the spatial and temporal change of the sensor signal distribution over the cell surface as the adhesion process advances. This information is erased in the aggregation. This supports the approach that cells should be processed as a whole, so that models can learn from the local features of the sensor data about how cells adhere to a specific surface. In these results, too, the AUC metric still shows higher values, which reinforces the assumption that this lack of variation is caused by the imbalance in the test set.
